# Vanadium pentoxide induces pulmonary inflammation and tumor promotion in a strain-dependent manner

**DOI:** 10.1186/1743-8977-7-9

**Published:** 2010-04-12

**Authors:** Elizabeth A Rondini, Dianne M Walters, Alison K Bauer

**Affiliations:** 1 Department of Pathobiology and Diagnostic Investigation and Center for Integrative Toxicology, Michigan State University, East Lansing, MI, 48824, USA; 2 Department of Physiology, Brody School of Medicine, East Carolina University, Greenville, North Carolina, 27834, USA

## Abstract

**Background:**

Elevated levels of air pollution are associated with increased risk of lung cancer. Particulate matter (PM) contains transition metals that may potentiate neoplastic development through the induction of oxidative stress and inflammation, a lung cancer risk factor. Vanadium pentoxide (V_2_O_5_) is a component of PM derived from fuel combustion as well as a source of occupational exposure in humans. In the current investigation we examined the influence of genetic background on susceptibility to V_2_O_5_-induced inflammation and evaluated whether V_2_O_5 _functions as a tumor promoter using a 2-stage (initiation-promotion) model of pulmonary neoplasia in mice.

**Results:**

A/J, BALB/cJ (BALB), and C57BL/6J (B6) mice were treated either with the initiator 3-methylcholanthrene (MCA; 10 μg/g; i.p.) or corn oil followed by 5 weekly aspirations of V_2_O_5 _or PBS and pulmonary tumors were enumerated 20 weeks following MCA treatment. Susceptibility to V_2_O_5_-induced pulmonary inflammation was assessed in bronchoalveolar lavage fluid (BALF), and chemokines, transcription factor activity, and MAPK signaling were quantified in lung homogenates. We found that treatment of animals with MCA followed by V_2_O_5 _promoted lung tumors in both A/J (10.3 ± 0.9 tumors/mouse) and BALB (2.2 ± 0.36) mice significantly above that observed with MCA/PBS or V_2_O_5 _alone (*P *< 0.05). No tumors were observed in the B6 mice in any of the experimental groups. Mice sensitive to tumor promotion by V_2_O_5 _were also found to be more susceptible to V_2_O_5_-induced pulmonary inflammation and hyperpermeability (A/J>BALB>B6). Differential strain responses in inflammation were positively associated with elevated levels of the chemokines KC and MCP-1, higher NFκB and c-Fos binding activity, as well as sustained ERK1/2 activation in lung tissue.

**Conclusions:**

In this study we demonstrate that V_2_O_5_, an occupational and environmentally relevant metal oxide, functions as an *in vivo *lung tumor promoter among different inbred strains of mice. Further, we identified a positive relationship between tumor promotion and susceptibility to V_2_O_5_-induced pulmonary inflammation. These findings suggest that repeated exposures to V_2_O_5 _containing particles may augment lung carcinogenesis in susceptible individuals through oxidative stress mediated pathways.

## Background

Lung cancer is the leading cause of cancer mortality in the U.S. and worldwide [1]. Although cigarette smoke is the main risk factor for lung cancer development, approximately 10-15% of cases occur in never-smokers, implicating other important environmental, occupational, and/or genetic factors [2-4]. Epidemiology studies have suggested that long-term exposure to elevated levels of particulate air pollution increases the risk of and mortality due to lung cancer [5-8]. Particulate matter (PM) is a complex mixture of particles that vary in physiochemical properties and are further classified according to the aerodynamic size (PM_2.5 _= <2.5 μm; PM_10 _= ≤10 μm) [9,10]. PM_2.5 _consists primarily of combustion products derived from automobiles and the burning of coal, fuel oil, and wood [9]. Most adverse health effects have been attributed to this fraction, due to the ability to penetrate deep within the alveolar region of the lung [11]. Using models developed by the World Bank, Cohen *et. al. *[12] predicted that 5% of respiratory cancer mortality worldwide is due to PM_2.5_.

The mechanism(s) contributing to increased lung cancer risk by PM have not been fully characterized, although it has been suggested that pulmonary inflammation mediated by particle-induced oxidative stress may play an important role [13,14]. Generation of reactive oxygen and nitrogen species (ROS/RNS) either directly or through activation of phagocytes can cause oxidative damage to DNA leading to initiation of cancer [14]. Additionally, ROS may potentiate tumor development by stimulating production of pro-inflammatory mediators that can promote expansion of initiated cells by influencing cell proliferation and apoptosis [14]. Oxidative stress induced by PM is dependent on both the surface area of the particle as well as its chemical composition [15]. Transition metals, and in particular vanadium compounds, have been implicated as the active constituents meditating oxidative lung injury in rodents exposed to residual fly oil ash (ROFA) [16-18] as well as in some studies using concentrated ambient air particles [19].

Vanadium pentoxide (V_2_O_5_) is the most common commercial form of vanadium [20]. V_2_O_5 _is released into the environment during oil and coal combustion and from metallurgical works [20]. Occupational exposure can be significant in the petrochemical, mining, and steel industries [20]. Additionally, military personnel and the general public can be exposed to high levels of vanadium as a result of incidental or intentional burning of fuel oils, such as exposures that occurred during the Kuwait oil fires in 1991 [21]. Adverse respiratory effects have been reported in humans, primates, and rodents exposed acutely to V_2_O_5_. Coughing, wheezing, chest pain, bronchitis, and asthma-like symptoms as well as impaired lung function occurred in humans exposed to high levels of V_2_O_5_-containing dust [22-25]. In primates, inhalation of V_2_O_5 _particles increased bronchoaveolar polymorphonuclear neutrophils (PMNs) and impaired pulmonary function [26], and in rodents, inhalation or intratracheal administration induced PMN influx, synthesis of pro-inflammatory mediators, as well as pulmonary fibrosis [27-30].

Occupational and ambient exposure to vanadium has been associated with an increase in biological markers for oxidative DNA damage [31,32], however limited data are available evaluating an association between V_2_O_5 _exposure on lung cancer risk [33,34]. *In vitro *studies suggest that vanadium functions as both an initiator and promoter of morphological transformation in cultured cell lines [35]. In a National Toxicology Program (NTP) study, continuous inhalation of V_2_O_5 _(24 months inhalation; 1-4 mg/m^3^) resulted in a significant increase (~50%) in the incidence of alveolar/bronchiolar neoplasms in both male and female B6C3F1 mice [30]. Although this study demonstrated the carcinogenic potential of V_2_O_5_, long-term continuous exposure was required before tumors developed and no dose response was observed, which suggests V_2_O_5 _may be promoting spontaneous tumors. In addition, different mouse strains were not assessed, which can greatly influence pulmonary responses to environmental pollutants [36] as well as susceptibility to carcinogenesis [37,38].

This study was conducted to further evaluate the role of V_2_O_5 _on pulmonary neoplasia among different inbred strains of mice. Using a two-stage (initiation-promotion) model, we hypothesized that inflammation induced by sub-chronic V_2_O_5 _administration would promote tumorigenesis in susceptible strains. Three strains of mice were included in this study that display altered susceptibility to chemical carcinogenesis: A/J mice are sensitive, BALB are intermediate, whereas B6 mice are resistant to most short term chemically-induced carcinogenesis protocols (*eg*. not initiatable using MCA) [37-39]. These same three strains also have similar differential susceptibility in chronic pulmonary inflammation models [40-42]. Results from this study demonstrate that V_2_O_5 _functions as an *in vivo *lung tumor promoter in both A/J and BALB mice. Further, we demonstrate a positive relationship between tumor promotion and susceptibility to V_2_O_5_-induced inflammation, involving the induction of the chemokines KC and MCP-1, the transcription factors NFκB and c-Fos, as well as sustained activation of ERK1/2 in pulmonary tissue.

## Methods

### Animal husbandry

Male A/J, BALB/cJ (BALB), C57BL/6J (B6) mice were purchased from Jackson Laboratories (Bar Harbor, ME) at 5-6 weeks of age. Animals were housed in plastic, filter-capped cages containing hardwood bedding and maintained in temperature (23 ± 2°C) and humidity (40-60%) controlled rooms with a 12 hour light/dark cycle. Animals were given standard laboratory chow (Teklan foods, Indianapolis, IN) and spring water *ad libitum *and were assessed daily for health status. All mice were allowed one week to acclimatize prior to treatment. Animal use was conducted in AAALAC-accredited facilities and in accordance with the regulatory guidelines of the Michigan State University All University Committee on Animal Use and Care.

### Preparation and Administration of Vanadium Pentoxide

**Figure 1:**
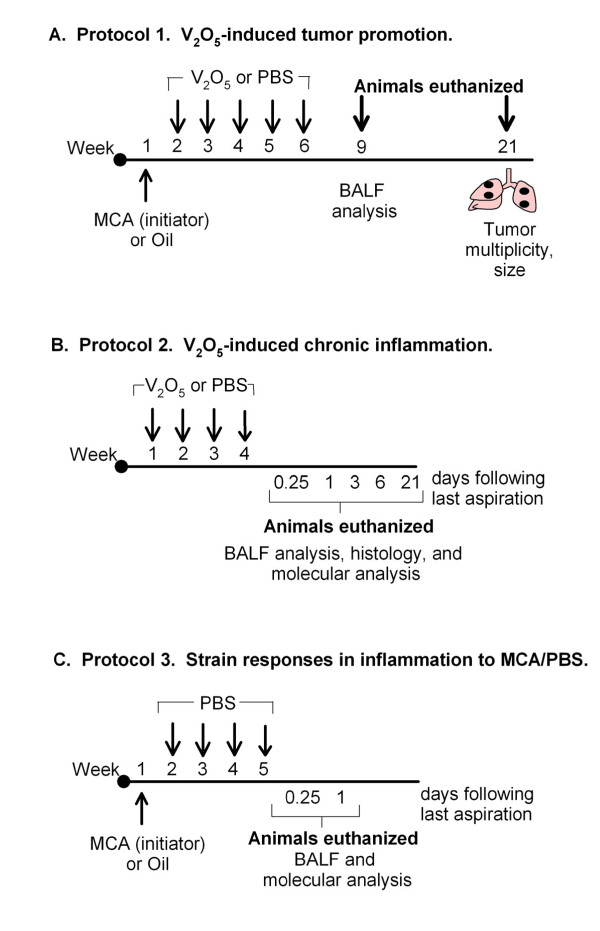
**Experimental protocols**. A.) V_2_O_5 _was evaluated as a tumor promoter using a two-stage carcinogenesis model. MCA suspended in corn oil (10 μg/g) was administered to initiate carcinogenesis followed by 5 weekly aspirations of V_2_O_5 _(4 mg/kg, promoter) or PBS. B.) A/J, BALB, and B6 mice were exposed to 4 weekly aspirations of V_2_O_5 _and sacrificed at select time points to assess pulmonary inflammation. C.) A/J, BALB, and B6 mice were administered MCA (10 μg/g) or oil and then exposed to 4 weekly aspirations of PBS and sacrificed at select time points to assess pulmonary inflammation.

Pulmonary administration of vanadium pentoxide (>99.9%, Sigma-Aldrich, St. Louis, MO) was performed by oropharyngeal aspiration as previously described [43]. Briefly, V_2_O_5 _was suspended in sterile-filtered Dulbecco's phosphate buffered saline (10 mM PBS, pH 7.4), sonicated for 20 minutes, then further diluted to a working concentration of 2 mg/mL. Prior to aspirations mice were anesthetized using 3% isoflurane in 1-2 L/min oxygen, and V_2_O_5 _(4 mg/kg body weight) was administered following the methods of Foster *et. al. *[43]. This dose was chosen based on preliminary dose response studies using protocol 2 (Fig. 1B) described below (data not shown), as well as previous acute lung injury models [28,29]. Control animals received PBS alone (50 μL/mouse). V_2_O_5 _suspensions were prepared fresh prior to use and administered to animals within two hours of preparation.

### Experimental Procedures

The experimental designs utilized in this study are depicted in Figure 1. Protocol 1 (Fig. 1A) was conducted to investigate whether sub-chronic exposure to V_2_O_5 _would promote pulmonary carcinogenesis using a two-stage (initiation-promotion) model. Mice were injected ip. (10 μg/g body weight) with the carcinogen MCA (Sigma, St. Louis, MO) dissolved in corn oil or with corn oil alone. Beginning one week later, mice were treated with 5 weekly aspirations of either V_2_O_5 _(4 mg/kg) or PBS as described above. To assess tumor promotion, animals were sacrificed 20 weeks following MCA treatment; the lungs were perfused with saline then inflated and fixed in Tellyesniczky's fixative for 48 hrs. Tumors were enumerated using an Olympus SZX7 stereomicroscope (Olympus; Center Valley, PA) and sized with digital calipers (Mitutoyo Corporation; Japan). Using this protocol, pulmonary inflammation was additionally assessed in A/J mice 21 days following the last aspiration as described below.

To assess strain differences in inflammation, (protocol 2, Fig. 1B), mice were aspirated once per week for 4 weeks with V_2_O_5 _(4 mg/kg) or PBS. At selected time intervals (6 hr, 1, 3, 6, and 21 days) following the last dose, bronchoaveolar lavage fluid (BALF) was collected to quantify differences in cellular infiltrates and protein content, a marker of hyperpermeability, as described previously [44]. At each time point, the right lobes were snap frozen in liquid nitrogen and stored at -80°C and the left lobe was either snap frozen and stored or inflated with and fixed overnight in 10% neutral buffered formalin for histological examination.

Because several studies demonstrated that MCA can induce p38 MAP Kinase, intracellular oxidants, as well as transcription factor activity in HepG2 cells (a hepatoma cell line) [45-47], an additional control experiment was conducted to determine whether carcinogen (MCA) administration influences pulmonary inflammation between strains. Mice were injected ip. with MCA (10 μg/g) dissolved in corn oil or oil alone, then aspirated with 4 weekly doses of PBS (Protocol 3, Fig. 1C) and sacrificed 6 hr or 1 day following the last aspiration. BALF was assessed for protein content and cellular infiltrate as described above.

### Immunohistochemical Detection of PMNs

A neutrophil-specific marker (sc-59338) and ABC detection kit (sc-2019) were purchased from Santa Cruz Biotechnology (Santa Cruz, CA). Left lungs were fixed in 10% NBF for 24 hrs, processed using standard histological procedures, embedded, then cut into 5 μm sections. Strain differences in pulmonary neutrophil infiltration were evaluated using peroxidase biotin-streptavidin immunohistochemistry, and bound enzyme was visualized using the chromagen 3-3'-diaminobenzidine (DAB). Tissues were then lightly counterstained in Gill's hematoxylin.

### Analysis of the chemokines KC, MIP-2, and MCP-1 by ELISA

ELISA kits for keratinocyte-derived chemokine (KC, CXCL1), macrophage inflammatory protein-2 (MIP-2, CXCL2), and monocyte chemoattractant protein 1 (MCP-1, CCL2) were purchased from R&D systems (Minneapolis, MN). Protein was prepared by homogenizing lungs in ice-cold RIPA buffer (10 mM PBS, 0.5% SDS, 0.5% sodium deoxycholate) containing protease inhibitors (Sigma, St. Louis, MO). Homogenates were centrifuged at 13,000 × *g *for 10 min at 4°C, and protein was quantified using the DC protein assay (BioRad; Carlsbad, CA). For chemokine analysis 25-50 μg of RIPA extracted protein was used in accordance with manufacturer's instructions. Absorbance was measured at 450 nm using a VersaMax microplate reader (Molecular Devices, Sunnyvale, CA). All data are presented as pg/mg protein.

### Transcription factor assay for nuclear NFκB and c-Fos activity

Nuclear protein was prepared from the left lung of mice using the TransAM nuclear extraction kit (Active Motif; Carlsbad, CA) and quantified with the DC protein assay (Biorad; Carlsbad, CA). Strain differences in binding of NFκB (p65 subunit) and AP-1 (c-Fos) were then measured from 8 μg of nuclear protein using TransAM Transcription Factor ELISA kits (Active Motif; Carlsbad, CA). Absorbance was measured at 450 nm using a VersaMax microplate reader (Molecular Devices, Sunnyvale, CA).

### Immunoblotting analyses for MAPK activation

Primary antibodies specific for MAPKs were purchased from Cell Signaling (Danvers, MA) and secondary antibodies from Pierce (Thermo Fisher; Rockford, IL). Protein was prepared from right lungs as described above. Samples (100 μg protein) were resolved on 12.5% SDS polyacrylamide gels. Following transfer, PVDF membranes were blocked for 1 hr at room temperature in 5% nonfat dry milk, and then incubated with primary antibodies to detect phosphorylated ERK1/2, JNK1/2, or p38 overnight at 4°C. After washing, blots were incubated in secondary antibody linked to horseradish peroxidase for 1 hr at room temperature, and bands were detected using chemiluminescence. Images were captured using BioRad ChemiDoc illumination system (BioRad; Carlsbad, CA). Following detection, membranes were stripped in Restore stripping buffer (Thermo Fisher; Rockford, IL) then reprobed for total MAPK using procedures described above. Densitometry of bands were quantified with BioRad Quality One software and phosphorylated proteins were normalized to the respective total MAPK prior to statistical analyses.

### Statistical analyses

All statistical analyses were conducted using SAS statistical software (SAS institute version 8.2, Cary, North Carolina). Time- and strain- dependent changes in BALF protein, cellularity, chemokines, nuclear transcription factor activity, protein densitometry, and tumor multiplicity/size were analyzed using an analysis of variance (ANOVA). When statistical differences were detected (*P *< 0.05), comparisons of means were analyzed using the least significant difference (LSD) method. All data are presented as mean ± SEM.

## Results

### Sub-chronic administration of V_2_O_5 _promotes pulmonary tumorigenesis in A/J and BALB mice

Strain differences in tumor multiplicity and size are presented in Table 1. We found that V_2_O_5 _functions primarily as a lung tumor promoter in both A/J and BALB mice following a low dose of MCA given as an initiating agent (Table 1; *P *< 0.05). For both strains, tumor multiplicity was higher in V_2_O_5_-treated mice compared to MCA-treated, PBS controls (*P *< 0.05). Additionally, a significant difference in tumor multiplicity and size was observed between BALB and A/J in the MCA group (Table 1). In the absence of MCA, V_2_O_5 _exposure alone was not sufficient to initiate tumorigenesis. No tumors were detected in B6 mice in any of the experimental groups examined (data not shown). In A/J and BALB mice, tumors were further evaluated by histopathological analyses in a subset of animals. A majority of the tumors were found to be solid adenomas (80%) and the remaining papillary (20%), consistent with previous studies using MCA [48].

**Table 1:** Lung tumor multiplicity and size (in parenthesis) among inbred mice following sub-chronic V_2_O_5 _exposure.^a, b, c, d^

	Corn Oil (Control)		MCA-treated	
		
Strain	PBS	**V**_**2**_**O**_**5**_	PBS	**V**_**2**_**O**_**5**_
A/J	0.0 ± 0.0	0.50 ± 0.50	3.3 ± 0.75^#^ (0.72 ± 0.036)^#^	10 ± 1.4*^#^ (0.72 ± 0.032)^#^
BALB	0.0 ± 0.0	0.0 ± 0.0	0.78 ± 0.28 (0.49 ± 0.039)	2.2 ± 0.36* (0.63 ± 0.068)

^a ^A/J, BALB, and B6 mice were treated with or without the initiator MCA (10 μg/g), followed by sub-chronic administration of V_2_O_5 _(4 mg/kg) or PBS.

^b ^Number of mice per treatment group: A/J, Oil/PBS and Oil/V_2_O_5 _(n = 3), MCA/PBS (n = 4), MCA/V_2_O_5 _(n = 15); BALB, Oil/PBS and Oil/V_2_O_5 _(n = 3-5), MCA/PBS (n = 8), MCA/V_2_O_5 _(n = 13); B6, Oil/PBS and Oil/V_2_O_5 _(n = 3-8), MCA/PBS (n = 7), MCA/V_2_O_5 _(n = 12).

^c ^No tumors were detected in B6 mice in any of the experimental groups examined (data not shown).

^d ^A significant strain effect was detected for tumor size (mm) between A/J (0.72 ± 0.012) and BALB mice (0.59 ± 0.054) (*P *< 0.05).

* Significantly different from strain-matched MCA-treated/PBS controls (*P *< 0.05).

# Significantly different than treatment-matched BALB mice (A/J *vs *BALB, *P *< 0.05).

### A/J and BALB mice are more susceptible to V_2_O_5_-induced pulmonary hyperpermeability and inflammation than B6 mice

**Figure 2:**
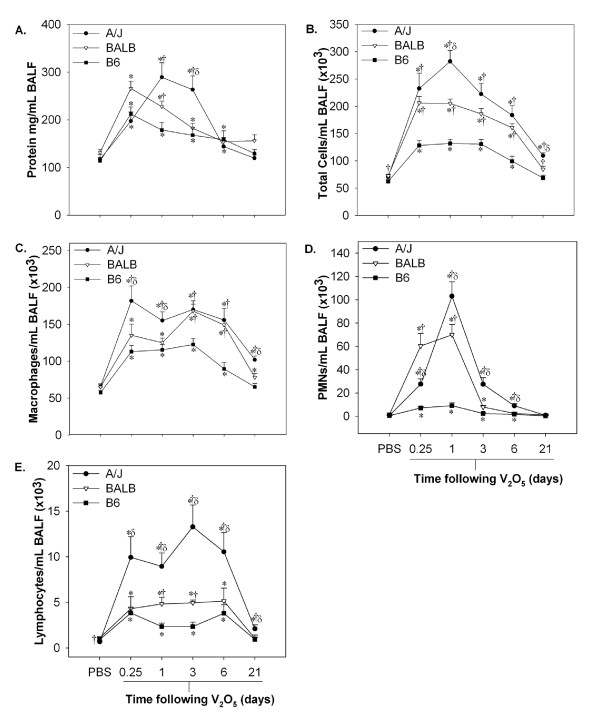
**A/J and BALB are susceptible to pulmonary inflammation and hyperpermeability in response to sub-chronic V_2_O_5_**. A/J, BALB, and B6 mice were exposed to 4 weekly doses of V_2_O_5 _(4 mg/kg) by aspiration and sacrificed 0.25, 1, 3, 6, 21 days after the last exposure. A.) Bronchoalveolar lavage (BALF) protein (μg/mL) and B.) total cells, C.) macrophages, D.) PMNs, and (E) lymphocytes per mL of BALF. Data represent the mean ± SEM (n = 5-15 animals/group). *, significantly different than strain-matched PBS controls. †, significantly different than time-matched B6 mice. δ, significantly different than time-matched BALB mice (*P *< 0.05).

To determine whether chronic inflammation was associated with tumor promotion, we evaluated strain differences in BALF cellularity and protein content up to 21 days following the final V_2_O_5 _dose (Fig. 2). In general, susceptibility to pulmonary inflammation and hyperpermeability proceeded in the order A/J>BALB>B6 mice (Fig. 2A-2E). BALF protein content increased significantly in all strains at 6 hr following V_2_O_5 _exposure and returned to baseline by 21 days (Fig. 2A, *P *< 0.05). The peak protein response was at 6 hr in BALB and B6 mice compared to 1 day in the A/J strain. Furthermore, protein levels in A/J mice remained significantly elevated above other strains at 3 days.

The effects of V_2_O_5 _on BALF cellularity are depicted in Fig. 2B-2E. As shown, the extent and duration of the inflammatory response was significantly greater in A/J mice at all time points examined (*P *< 0.05; Fig. 2B). The most striking difference between strains was observed for PMNs, which was highest at 1 day (Fig. 2D). A/J mice exhibited a ~150-fold increase in the number of PMNs infiltrating the lung representing 36% of the total cells recovered compared to a 43-fold increase in BALB (34%) and only a 16-fold increase (~7%) in B6 mice. By 21 days, inflammation completely resolved in B6 mice, but the total number of cells, primarily macrophages and some lymphocytes, remained elevated in A/J and to a lesser degree in BALB mice (*P *< 0.05).

**Figure 3:**
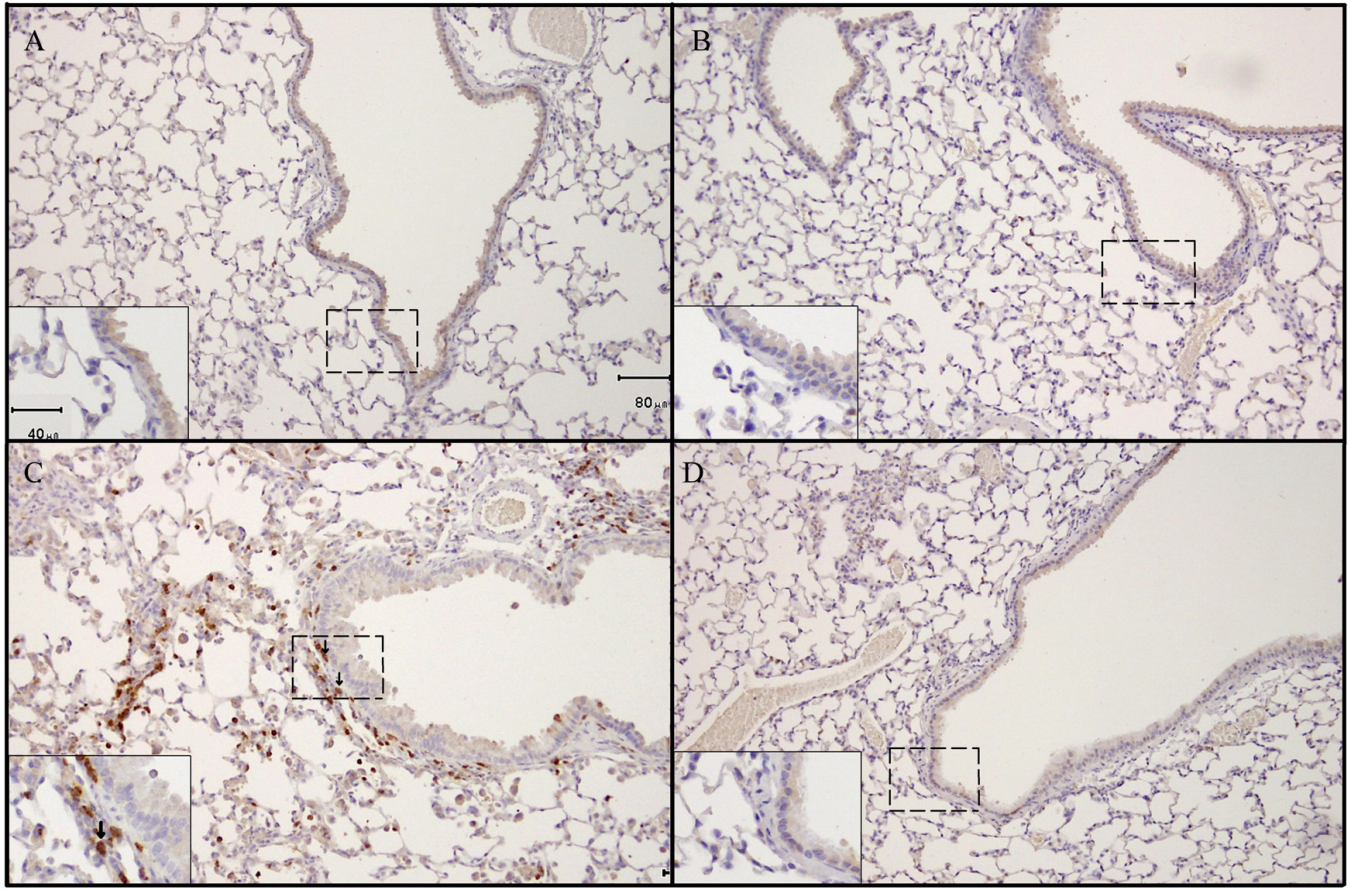
**Neutrophil influx into lungs of A/J mice in response to sub-chronic V_2_O_5_**. Neutrophils were detected by immunohistochemical staining using an anti-mouse neutrophil antibody in formalin fixed lung sections. A.) PBS-treated A/J mouse; B.) PBS-treated B6 mouse, C.) V_2_O_5_-treated A/J mouse, 1 day; D.) V_2_O_5_-treated B6 mouse, 1 day. Original magnifications ×100, insets ×200. Arrows indicate areas with positive staining for neutrophils (red-brown in color), dashed boxes indicate areas magnified within the insets.

The BALF results were further confirmed using histological staining with an anti-PMN marker in lung sections from the most (A/J) and least sensitive (B6) strains 1 day following the final V_2_O_5 _dose (Figure 3). Figures 3A-D demonstrate higher PMN influx in the A/J strain compared to both B6 mice and PBS controls. Positive staining for PMNs was observed primarily around the bronchioles and in close proximity to the alveolar epithelium, although staining was also seen surrounding larger airways. Increased cellularity of the bronchiolar and alveolar epithelium, indicative of epithelial cell proliferation, was also observed in A/J, but not B6 mice when compared to PBS controls.

In a separate study, we assessed the inflammatory cell profile in the most sensitive strain (A/J) 21 days following the last V_2_O_5 _dose to determine any synergistic effect of MCA on inflammation (Table 2). For all phenotypes examined (BALF total protein, and total cells including macrophages, lymphocytes, and PMNs), there were significant increases in mice treated with V_2_O_5 _compared to PBS controls. There were no significant differences between animals treated with MCA or with oil in any of the groups (Table 2), suggesting that V_2_O_5 _was primarily driving the inflammatory response. The total cell numbers and macrophages in this study were higher compared to Protocol 2 at this time point (21 days), due to the extra weekly dose of V_2_O_5 _used to maximize promotion.

**Table 2:** Pulmonary inflammation and hyperpermeability in A/J mice treated with either corn oil or MCA (10 μg/g) and then aspirated with 5 weekly doses of V_2_O_5 _(4 mg/kg) or PBS.^a^

Treatment	Protein (μg/mL)	**Total Cells (×10**^**3**^**)**	**Macrophages (×10**^**3**^**)**	**Lymphocytes (×10**^**3**^**)**	PMNs **(×10**^**3**^**)**
Oil/PBS	129 ± 4.3	79.7 ± 8.8	73.7 ± 7.9	0.572 ± 0.12	0.422 ± 0.12
Oil/V_2_O_5_	166 ± 4.0*	156 ± 17*	147 ± 18*	4.14 ± 0.65*	0.737 ± 0.23*
MCA/PBS	132 ± 15	83.1 ± 11	76.3 ± 11	0.109 ± 0.07	0.271 ± 0.073
MCA/V_2_O_5_	198 ± 25*	161 ± 16*	149 ± 18*	6.01 ± 0.81*	1.47 ± 0.38*

^a ^A/J mice were treated with corn oil (control) or MCA (10 μg/g) and then aspirated with 5 weekly doses of either PBS or V_2_O_5 _(4 mg/kg). Animals were sacrificed 21 days after the last aspiration. Protein (μg/mL) concentration and inflammation were measured in bronchoalveolar lavage fluid (BALF) and data are expressed as cells (×10^3^) per mL of BALF. Data represent the mean ± SEM (n = 4-7 animals/group).

* Significantly different from PBS exposed animals (*P *< 0.05).

To further confirm that strain differences in tumor promotion were not due to differences in inflammatory responses to MCA, an additional control experiment was conducted (Fig. 1C, Protocol 3). MCA or oil was administered to mice followed by 4 weekly doses of PBS and differences in BALF protein content and cellularity were measured at 6 hr and 1 day following the last aspiration (Additional file 1, Table S1). BALB mice exhibited a significant increase in protein levels compared to the other strains, similar to that observed in Fig. 2. Both BALB and A/J mice also had higher PMNs compared to B6 mice, however no additional effects of MCA on inflammatory cell types were observed within strains (Additional file 1, Table S1). Thus, these results provide further evidence that strain susceptibility to inflammation induced by V_2_O_5 _and not to MCA is more strongly associated with lung tumor promotion in our model.

### Strain differences in V_2_O_5_-induced inflammatory chemokine (KC, MIP-2, MCP-1) expression

**Figure 4:**
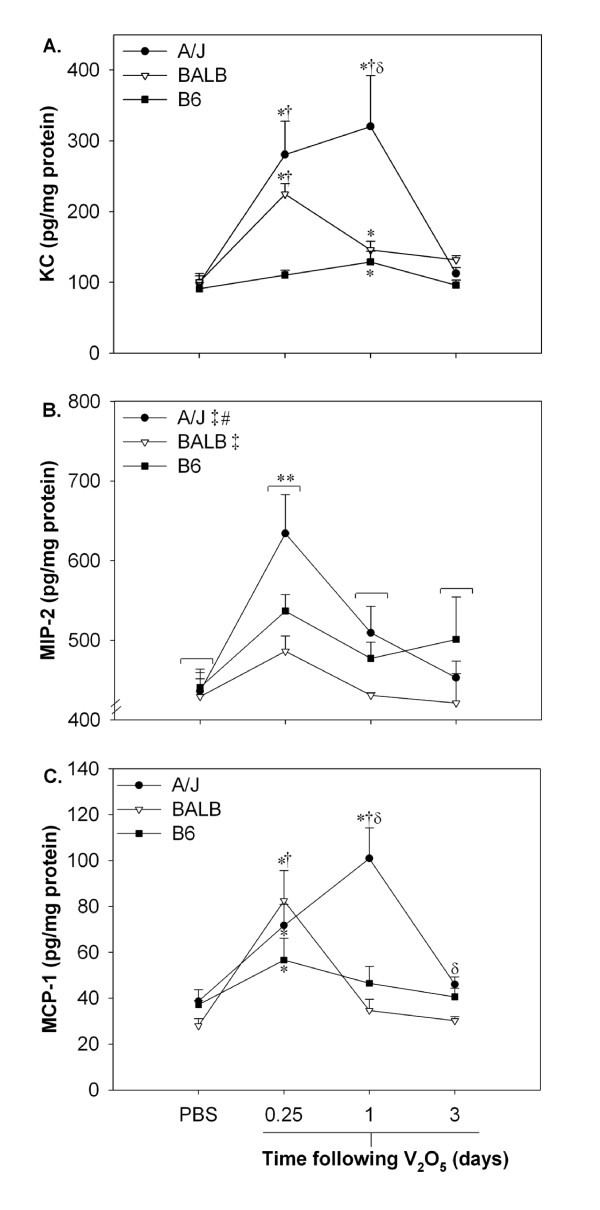
**The chemokines KC and MCP-1 are elevated in A/J and BALB mice following V_2_O_5 _treatment**. A.) KC, keratinocyte chemoattractant, B.) MIP-2, macrophage inflammatory protein-2, C.) MCP-1, monocyte chemoattractant protein-1 levels were determined in lung homogenates (25-50 μg protein) by ELISA. Values are presented as means ± SEM from two independent assays. *, significantly different than strain-matched PBS controls. †, significantly different than time-matched B6 mice. δ, significantly different than time-matched BALB mice. **, significant treatment effect (6 hr V_2_O_5 _*vs *PBS control). ‡, significant strain effect (A/J *vs *B6, BALB *vs *B6). #, significant strain effect (A/J *vs *BALB) (*P *< 0.05).

Differential strain responses were detected for the chemokines KC and MCP-1 (Fig. 4A, C), with higher levels observed in both A/J and BALB compared to B6 mice. KC increased 3-fold and MCP-1 by 2.5-fold at 6 hr and remained elevated in A/J mice at 1 day following the last V_2_O_5 _dose (Fig. 4A, C; *P *< 0.05). In BALB mice, chemokine levels increased to a similar extent at 6 hr, but levels declined sharply by 1 day. Comparably, only modest increases (~1.5-fold) were seen in B6 mice. Strain responses were more variable and less pronounced for MIP-2 (Fig. 4B). MIP-2 increased significantly in all strains at 6 hr, with overall changes of 1.5-fold in A/J, 1.1-fold in BALB, and 1.2-fold in B6 mice.

### A/J mice have higher transcriptional activity of NFκB and AP-1 than B6 mice following V_2_O_5_

**Figure 5:**
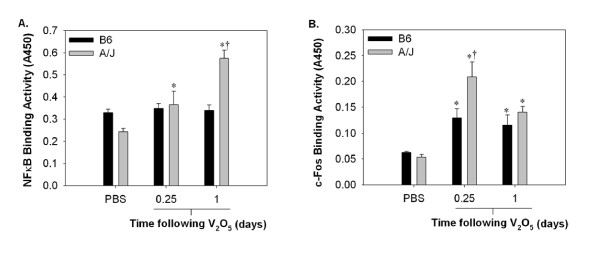
**NFκB and c-Fos are differentially regulated in A/J and B6 mice following V_2_O_5 _instillation**. Nuclear binding activity for A.) NFκB and B.) c-Fos were determined by transcription factor ELISA TransAM kits in nuclear protein extracts (8 μg) prepared from the lungs of chronic V_2_O_5 _treated mice (n = 3-6/group). *, significantly different than strain-matched PBS controls (*P *< 0.05). †, significantly different than time-matched B6 mice (*P *< 0.05).

Nuclear transcription factor activity and MAPK signaling (see below) were evaluated in the most sensitive (A/J) and resistant (B6) strains (Fig. 5). Sub-chronic administration of V_2_O_5 _resulted in higher nuclear NFκB binding activity in A/J mice at both 6 hr and 1 day, with binding activity at 1 day significantly greater than all other groups (Fig. 5A; *P *< 0.05). No corresponding changes were observed in B6 mice at the time points assessed (Fig. 5A). Nuclear activity for the AP-1 transcription factor, c-Fos increased significantly in both strains, but more so at 6 hr in A/J compared to B6 mice (Fig. 5B; *P *< 0.05).

### V_2_O_5 _activates the MAPKs ERK1/2 and p38 in pulmonary tissue

**Figure 6:**
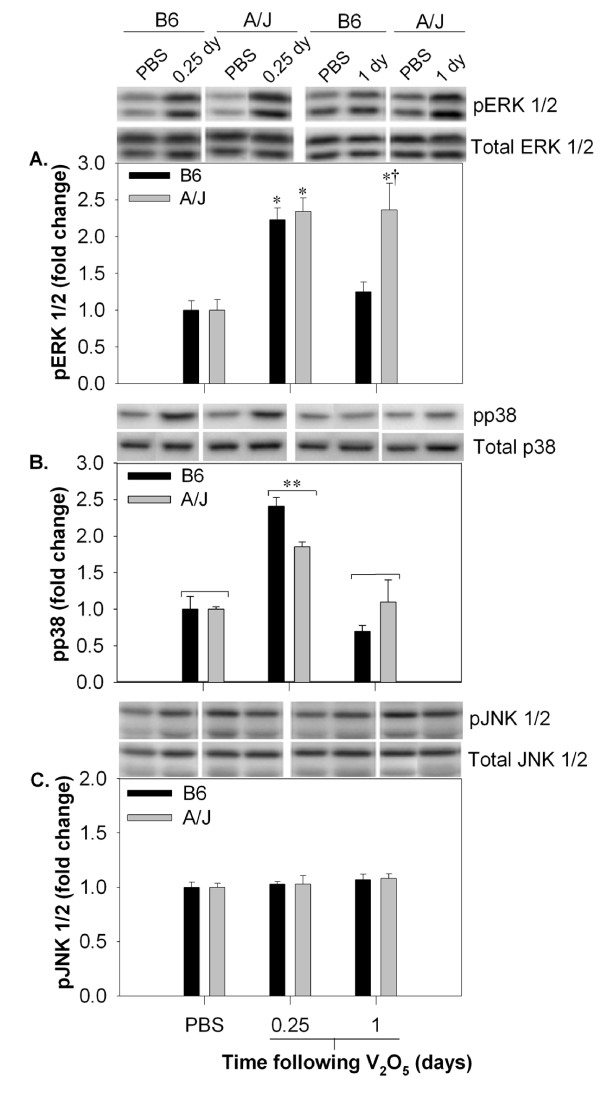
**ERK1/2 is differentially activated in A/J compared to B6 mice following sub-chronic V_2_O_5 _instillation**. Homogenates were prepared from the right lungs of mice A.) 6 hr and B.) 1 day after the last V_2_O_5 _exposure (n = 3-5/group). Phosphorylated and total levels of MAPK in lung homogenates were analyzed from 100 μg protein by Western blotting. Representative images and mean band intensities are representative of 2-3 independent experiments. *, significantly different than strain-matched PBS controls (*P *< 0.05). **, significant main effect of time. †, significantly different than time-matched B6 mice (*P *< 0.05).

Strain differences in MAPK signaling were assessed in whole lung homogenates (Fig. 6). Compared to PBS controls, a significant increase in phosphorylation of p38 and ERK1/2 were observed in both A/J and B6 mice 6 hr following V_2_O_5 _treatment. By one day, phospho-p38 returned to basal levels in both strains (Fig. 6B), whereas phospho-ERK1/2 remained elevated in A/J mice (Fig. 6A; *P *< 0.05). No significant changes in JNK1/2 were observed between or within strains at any of the time points examined (Fig. 6C). Because MCA has been shown to induce intracellular oxidant levels [46], we also measured the MAPKs ERK1/2 and p38 in B6 and A/J mice from Protocol 3 (Additional file 2, Fig. 1S). There was a slight but non-significant increase in phospho-p38 in MCA-treated B6 mice 6 hr following the last PBS aspiration, however no significant differences between strains were observed for either MAPK examined (Additional file 2, Fig. 1S).

## Discussion

Chronic inflammation is a risk factor for several cancer types [49]. Asthmatics and individuals with COPD are at an elevated lifetime risk for developing lung cancer [50]. The importance of inflammation in augmenting pulmonary carcinogenesis is further supported by a wide range of pharmaceutical compounds that inhibit neoplastic development [51] as well as evidence from transgenic mouse models [52,53]. Because tumor promotion involves changes in gene expression, most likely epigenetic in nature, and is the only reversible stage of carcinogenesis, studying promoters may identify additional pathways to target for preventive strategies against human lung cancer.

In the current investigation, we provide evidence that V_2_O_5 _functions as an *in vivo *tumor promoter among differentially susceptible inbred strains of mice. Using a two-stage model of carcinogenesis, a significant increase in tumor multiplicity was observed in both A/J (10.3 ± 0.9 tumors/mouse) and BALB (2.2 ± 0.36) mice exposed to the carcinogen MCA followed by 5 weekly aspirations of V_2_O_5_. The effect of V_2_O_5 _was limited to tumor promotion, as no significant increase in tumor numbers were observed in animals exposed to V_2_O_5 _alone. Susceptibility to promotion paralleled relative strain sensitivity to V_2_O_5_-induced inflammation: A/J mice were most sensitive and BALB were intermediate. B6 mice were found to be most resistant to V_2_O_5_-induced inflammation, however were used as a control since they are not initiated by the low dose of MCA administered in this study [37].

Differences between the two susceptible strains of mice (A/J and BALB) are not unusual based on past genome mapping studies demonstrating distinct genes responsible for tumorigenesis in these specific strains [54,55]. While both strains are susceptible to lung tumor development, differences in sensitivity between these two strains has been linked to quantitative trait loci containing both tumor suppressor genes as well as inflammatory mediators, such as myeloperoxidase (*Mpo*), colony stimulating factor *(Csf)3*, CC chemokine receptor (*Ccr10*), and *Ccl2 *(*Mcp-1*) [54,55]. Although MCA was used as an initiating agent in this study, additional control experiments further demonstrated that carcinogen treatment alone did not influence inflammatory indices between strains. Because significant strain responses were observed only in response to V_2_O_5_, our findings suggest that that genetic (host) factors contributing to V_2_O_5_-induced pulmonary inflammation are also strongly associated to lung tumor promotion.

Vanadium is thought to mediate pulmonary inflammation through generation of multiple reactive oxygen species (O_2_^-^, H_2_O_2_, and ·OH) in target cells [56-58]. Production of ROS is associated with phosphorylation of EGF-R and activation of MAPK signaling [57,59-63] as well as the transcription factors NFκB [59,63], AP-1 [59,64], and STAT-1 [65]. Furthermore, vanadium is known to be a phosphatase inhibitor [66] and likely prolongs phosphorylation and signaling along ROS-sensitive pathways. These events, in turn can influence the synthesis and release of pro-inflammatory cytokines and chemokines mediating acute lung injury [29,65,67,68]. Pretreatment of human bronchial epithelial cells with metal chelators and/or free radical scavengers reduces vanadium-generated ROS, MAPK activation, as well as release of chemokines, further supporting a role for oxidative stress in vanadium-induced inflammation [62].

In our study, differential strain induction of chemokines and upstream signaling molecules in response to V_2_O_5 _correlated to the extent and duration of inflammatory cells recovered in pulmonary tissue. MIP-2 and KC are principle neutrophil chemoattractants in rodent models, homologous to IL-8 in humans [69], whereas MCP-1 induces monocyte and lymphocyte chemotaxis and migration [70]. We observed moderate, although significant induction of MIP-2 in all strains at 6 hr following vanadium exposure, which likely involved initial PMN influx. However, strain differences in the peak PMN response were more closely associated with pulmonary levels of KC. MCP-1 was highly induced in A/J and BALB mice and expression coincided with the influx of both monocytes and lymphocytes into pulmonary tissue. The transcription factors NFκB and c-Fos as well as the MAPK pERK1/2 were also found to be differentially regulated in the sensitive (A/J) and resistant (B6) mice and corresponded to both altered chemokine induction and BALF cellularity.

The microenvironment is becoming increasingly recognized as actively contributing to the tumorigenic process. Evidence suggests that PMNs and macrophages appear to be involved in tumor development through multiple mechanisms, including more direct, such as induction of DNA damage and regulation of cell cycle [71], as well as indirect mechanisms, such as promotion of angiogenesis by cytokines and chemokines and suppression of adaptive immune responses [71,72]. Local production of cytokines and chemokines may also stimulate expansion of initiated cells by influencing cell proliferation and apoptotic pathways [14]. Several signaling molecules altered by V_2_O_5 _in this study have been implicated in lung cancer development. For example, IL-8 has been reported to serve as an autocrine growth factor in lung cancer cell lines [73,74] and both IL-8 and MCP-1 are elevated in bronchiolar epithelium from patients with COPD [75,76] and non-small cell lung cancer (NSCLC) [77]. In mouse models, neutralization of CXCR2, the principle receptor for KC and MIP-2 reduces PMN infiltration [78] as well as tumor growth and angiogenesis, suggesting a role in tumor progression [53,79,80]. Constitutive activation of pERK1/2 [81,82] and the transcription factors NFκB [83] and c-Fos [84] have well known effects on cell cycle regulation. Additional evidence for ERK1/2 in pulmonary tumorigenesis was recently demonstrated in transgenic mice overexpressing mutant *B-raf *and *K-ras*. Pharmacological inhibition of pERK1/2 resulted in tumor regression by inhibiting cell proliferation and restoring apoptosis [81]. Constitutive activation of ERK1/2 was also observed in V_2_O_5_-induced mouse carcinomas from the NTP study containing both *K*-*ras *mutations and loss of heterozygosity [85], which supports findings in this model and suggests involvement of ERK1/2 as one pathway driving tumor promotion by V_2_O_5_.

## Conclusions

Our study provides evidence that V_2_O_5 _functions as an *in vivo *tumor promoter and suggests that susceptibility to V_2_O_5_-induced inflammation and tumor promotion is influenced by genetic background. Tumor promotion in our model was associated with a robust inflammatory response involving induction of multiple chemokines, the transcription factors NFκB and c-Fos, as well as sustained activation of ERK1/2 in susceptible mice. These findings suggest that activation of oxidative stress-mediated signaling events may be one mechanism contributing to increased lung cancer risk by PM. A limitation in the current study was that the dose of V_2_O_5 _utilized was significantly higher than either occupational or ambient exposure levels, and was not meant to be directly used for risk assessment. It should be noted, however, that in the NTP study, a significant increase in pulmonary tumors was also reported after 2 years in B6C3F1 mice, a resistant strain, at more relevant occupational levels of V_2_O_5_. Although we found that V_2_O_5 _alone did not initiate tumorigenesis, our findings highlight that repeated exposures to inflammatory stimuli augments pulmonary carcinogenesis in susceptible strains. Additional studies examining genetic differences in antioxidant enzyme levels and adenoma susceptibility genes potentially contributing to tumor promotion by V_2_O_5 _as well as to other PM constituents warrant further investigation.

## Abbreviations

ANOVA: Analysis of variance; AP-1: Activator protein-1; BALB: BALBc/J; B6: C57BL/6J; BALF: Bronchoalveolar lavage fluid; COPD: chronic obstructive pulmonary disease; ELISA: Enzyme-linked immunosorbent assay; ERK: Extracellular-signal related kinase; KC: Keratinocyte-derived chemokine; MAPK: Mitogen-activated protein kinase; MCP-1: Monocyte chemoattractant protein-1; MIP-2: Macrophage inflammatory protein-2; NSCLC: non small cell lung cancer; NFκB: Nuclear factor-kappa B; JNK: c-Jun N-terminal kinase; PBS: Phosphate buffered saline; PM: Particulate matter; RNS: Reactive nitrogen species; ROS: Reactive oxygen species; ROFA: Residual oil fly ash; TBS: Tris buffered saline; TBST: Tris buffered saline with Tween-20; V_2_O_5_: Vanadium pentoxide.

## Competing interests

The authors declare that they have no competing interests.

## Authors' contributions

EAR performed V_2_O_5 _exposures, BAL analysis, and all experimental procedures (immunohistochemistry, ELISAs, immunoblots, transcription factor assays), as well as drafted the manuscript. DMW assisted in experimental design. AKB conceived of the study design and methodology utilized, assisted in V_2_O_5 _exposures and euthanasia, enumerated pulmonary tumors, and helped to draft the manuscript. All authors read and approved the final manuscript.

## Supplementary Material

Additional file 1 **Table S1. Pulmonary inflammation and hyperpermeability in B6, BALB, and A/J mice treated with corn oil or MCA (10 μg/g) and then aspirated with 4 weekly doses of PBS**. Click here for file

Additional file 2 **Figure S1. The MAPKs ERK 1/2 and p38 are not significantly altered between B6 or A/J mice treated with the carcinogen MCA (10 μg/g) and then aspirated with 4 weekly doses of PBS**. Homogenates were prepared from the right lungs of mice treated with either MCA or oil and then 4 weekly aspirations of PBS (n = 3-5/group). Phosphorylated and total levels of MAPK in lung homogenates were analyzed from 75 μg protein by Western blotting. Representative images and mean band intensities are representative of 2-3 independent experiments. No significant differences were observed for either of the MAPK tested (*P *> 0.05). Click here for file
