# Supplementary figures and images for: Vanadium pentoxide induces pulmonary inflammation and tumor promotion in a strain-dependent manner
Source: Part Fibre Toxicol. 2010 Apr 12;7:9. doi: 10.1186/1743-8977-7-9 (PMC2861012; doi:10.1186/1743-8977-7-9)

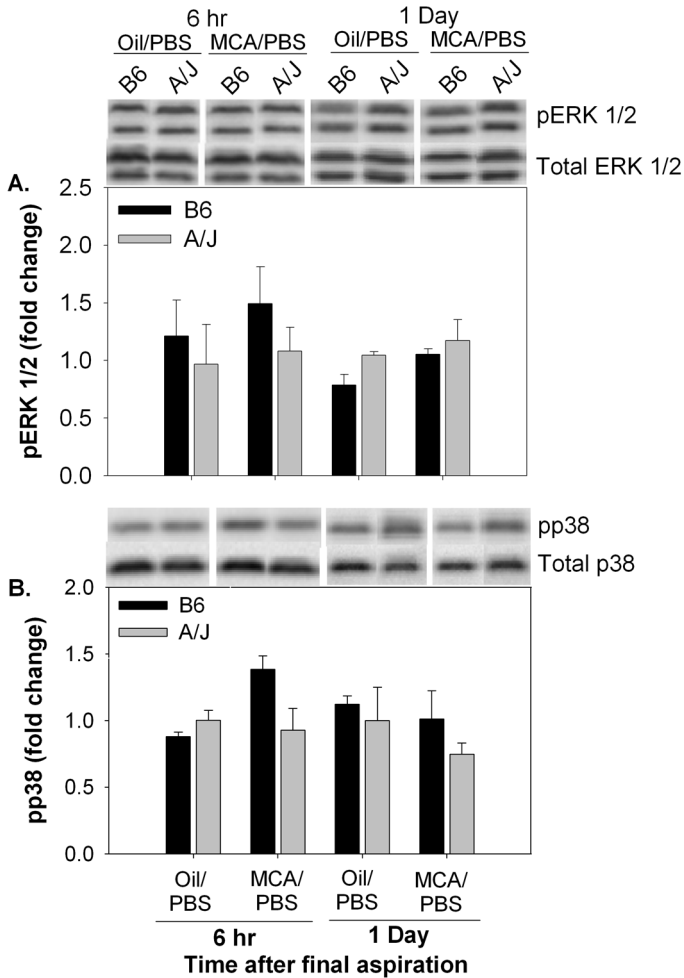

Supplement: Additional file 1 — Table S1. Pulmonary inflammation and hyperpermeability in B6, BALB, and A/J mice treated with corn oil or MCA (10 μg/g) and then aspirated with 4 weekly doses of PBS. [file 1743-8977-7-9-S1.PDF]
