# Supplementary material for: Vanadium pentoxide induces pulmonary inflammation and tumor promotion in a strain-dependent manner
Source: Part Fibre Toxicol. 2010 Apr 12;7:9. doi: 10.1186/1743-8977-7-9 (PMC2861012; doi:10.1186/1743-8977-7-9)
Supplement: Additional file 2 — Figure S1. The MAPKs ERK 1/2 and p38 are not significantly altered between B6 or A/J mice treated with the carcinogen MCA (10 μg/g) and then aspirated with 4 weekly doses of PBS. Homogenates were prepared from the right lungs of mice treated with either MCA or oil and then 4 weekly aspirations of PBS (n = 3-5/group). Phosphorylated and total levels of MAPK in lung homogenates were analyzed from 75 μg protein by Western blotting. Representative images and mean band intensities are representative of 2-3 independent experiments. No significant differences were observed for either of the MAPK tested (P > 0.05). [file 1743-8977-7-9-S2.DOC]

| **Additional File Table S1. Pulmonary inflammation and hyperpermeability in B6, BALB, and A/J mice treated with corn oil or MCA (10 µg/g) and then aspirated with 4 weekly doses of PBS.^a^** | | | | | | | |
| --- | --- | --- | --- | --- | --- | --- | --- |
|  |  |  | **BALF Protein (µg/mL)*** | **Total Cells**  **(x10^3^)*^#^*** | **Macrophages**  **(x10^3^)** | **Lymphocytes**  **(x10^3^)** | **PMNs**  **(x10^3^)^δ^** |
| B6 | Oil | 6 hr | 114 ± 2.7 | 57.5 ± 6.3 | 53.0 ± 5.1 | 0.31 ± 0.18 | 1.31 ± 0.83 |
|  | MCA |  | 128 ± 7.0 | 48.9 ± 6.6 | 45.6 ± 6.8 | 0.61 ± 0.044 | 1.38 ± 1.2 |
|  | Oil | 1day | 98.5 ± 6.2 | 43.8 ± 3.3 | 40.0 ± 3.4 | 0.61 ± 0.26 | 0.87 ± 0.58 |
|  | MCA |  | 115 ± 8.0 | 53.0 ± 5.2 | 47.7 ± 4.2 | 0.83 ± 0.12 | 1.10 ± 0.41 |
| BALB | Oil | 6 hr | 135 ± 8.7 | 56.3 ± 6.9 | 52.9 ± 7.5 | 0.52 ± 0.29 | 3.02 ± 1.9 |
|  | MCA |  | 135 ± 11 | 48.4 ± 4.7 | 42.7 ± 5.6 | 1.30 ± 0.15 | 2.55 ± 1.1 |
|  | Oil | 1 day | 114 ± 13 | 44.8 ± 3.3 | 39.6 ± 4.6 | 0.88 ± 0.35 | 2.25 ± 0.72 |
|  | MCA |  | 135 ± 8.5 | 53.0 ± 5.1 | 45.3 ± 6.3 | 0.94 ± 0.37 | 2.17 ± 0.59 |
| A/J | Oil | 6 hr | 120 ± 9.1 | 62.9 ± 0.42 | 56.7 ± 2.8 | 0.42 ± 0.00 | 2.29 ± 2.1 |
|  | MCA |  | 106 ± 8.8 | 57.0 ± 5.0 | 51.3 ± 5.3 | 0.40 ± 0.09 | 2.89 ± 2.6 |
|  | Oil | 1 day | 98.2 ± 2.4 | 50.6 ± 4.4 | 45.2 ± 3.7 | 0.72 ± 0.56 | 2.18 ± 0.94 |
|  | MCA |  | 106 ± 13 | 53.4 ± 3.9 | 46.5 ± 4.3 | 0.87 ± 0.25 | 1.69 ± 0.25 |
| **^a^** Mice were treated with corn oil (control) or MCA (10 µg/g) and then aspirated with 4 weekly doses of PBS. Animals were sacrificed 6 hr or 1day following the last aspiration. Protein (μg/mL) concentration and inflammation were measured in bronchoalveolar lavage fluid (BALF). Data are expressed as cells (x10^3^) per mL BALF and represent the mean ± SEM (n=3-7 animals/group).  * BALB mice had significantly higher levels of total BALF protein compared to A/J and B6 mice (P*<*0.05); # significant effect of treatment and time detected, with higher levels of total cells present in MCA treated animals 1 day following the last dose of PBS (*P*<0.05); ^#^ significant strain effect detected, with more PMNs recovered in BALF from BALB and A/J mice compared to the B6 strain (*P*<0.05). | | | | | | | |
